# CORRECTING GLUTATHIONE DEFICIENCY IN AGING: IMPACT ON MITOCHONDRIA, STRENGTH, INFLAMMATION AND METABOLIC DEFECTS

**DOI:** 10.1093/geroni/igz038.1550

**Published:** 2019-11-08

**Authors:** Rajagopal Sekhar, George E Taffet, Roger Fielding

**Affiliations:** 1 Baylor College of Medicine, Houston, Texas, United States; 2 Tufts University, Boston, Massachusetts, United States

## Abstract

Aging is associated with deficiency of Glutathione, the most abundant, intracellular, antioxidant protein, but underlying mechanisms are unknown and interventions limited. This symposium is primarily focused on the results of placebo-controlled, double-blind randomized clinical-trial (RCT) on the impact of correcting Glutathione deficiency in older humans on mitochondrial impairment, oxidative stress, strength, inflammation, and insulin resistance. Dr. Jahoor’s presentation will serve as an introduction by discussing mechanisms underlying Glutathione deficiency and validation of a novel nutritional intervention based on supplementing glycine and N-acetylcysteine (GlyNAC) to correct Glutathione deficiency in older-humans. Dr. Sekhar will present the results of a pilot 16-week pilot randomized, placebo-controlled, double-blind clinical trial in older humans investigating the effect of supplementing GlyNAC (vs. placebo) to improve Glutathione levels and oxidative-stress in 24 older-humans and 12 young-humans on impaired mitochondrial fuel-oxidation (MFO) and other defects. The trial met its primary objective that that GlyNAC supplementation (and not placebo) significantly improved Glutathione deficiency and corrected impaired MFO (and defects in its molecular regulation), and also significantly improved gait-speed (increased 19% increase to match young-humans), muscle-strength, exercise-capacity, and lowered oxidative-stress (80%) inflammation (IL-6 83%, TNF-alpha 58%), and insulin-resistance (68%). Dr. Taffet will discuss age-induced diastolic heart failure, and the effect of supplementing GlyNAC (vs. NAC alone) in aged 24-month old mice with diastolic heart-failure, impaired myocardial MFO and cardiac-inflammation. Collectively this symposium on Glutathione and Aging will highlight the discovery that supplementing GlyNAC to correct Glutathione deficiency in older-humans has significant health benefits, and could be a novel nutritional-intervention in aging.

